# Towards a systematic assessment of assay interference: Identification of extensively tested compounds with high assay promiscuity

**DOI:** 10.12688/f1000research.12370.2

**Published:** 2017-10-09

**Authors:** Erik Gilberg, Dagmar Stumpfe, Jürgen Bajorath

**Affiliations:** 1 Department of Life Science Informatics, B-IT, LIMES Program Unit Chemical Biology and Medicinal Chemistry, Rheinische Friedrich-Wilhelms-Universität, Bonn, D-53113, Germany

**Keywords:** Biological screening data, statistical analysis, active compounds, assay frequency, hit rate distribution, assay interference

## Abstract

A large-scale statistical analysis of hit rates of extensively assayed compounds is presented to provide a basis for a further assessment of assay interference potential and multi-target activities. A special feature of this investigation has been the inclusion of compound series information in activity analysis and the characterization of analog series using different parameters derived from assay statistics. No prior knowledge of compounds or targets was taken into consideration in the data-driven study of analog series. It was anticipated that taking large volumes of activity data, assay frequency, and assay overlap information into account would lead to statistically sound and chemically meaningful results. More than 6000 unique series of analogs with high hit rates were identified, more than 5000 of which did not contain known interference candidates, hence providing ample opportunities for follow-up analyses from a medicinal chemistry perspective.

## Introduction

Compounds with false-positive signals in biological assays cause substantial problems for biological screening and medicinal chemistry ^1^. Assay artifacts often remain undetected or are unveiled only at later stages of compound development efforts, leading to substantial loss of time and resources. Moreover, once published, artificial activities spread through the scientific literature and potentially cause even more harm by inspiring follow-up investigations that are doomed to fail. Known assay interference compounds include colloidal aggregators ^2– 7^ and many other compound classes that can react in different ways or are fluorescent under assay conditions ^6– 15^. Systematic efforts to identify interference compounds include the compilation of aggregators ^2– 4^ and pan-assay interference compounds (PAINS) ^8, 9^. The latter comprise a set of 480 classes of compounds originally identified in AlphaScreen assays ^8^. PAINS are typically contained as substructures in larger compounds. However, the assessment and prediction of assay interference is far from being a trivial exercise. For example, analysis of screening data from PubChem ^16^ has revealed that many compounds containing PAINS, including most reactive chemical entities, have very different hit rates or might be consistently inactive ^17, 18^. Moreover, analogs or different series of analogs containing the same PAINS substructure often have distinct activity profiles and are active against different targets ^19^. Thus, interference characteristics of related compounds frequently differ and a substructure with interference potential does not necessarily give rise to false-positive assay signals. To further complicate matters, promiscuous compounds may also have true multi-target activities ^20^ that are relevant for polypharmacology ^20– 22^. Moreover, even highly promiscuous screening hits include molecules with no apparent liabilities, in addition to obvious interference compounds ^12^.

Without doubt, judging assay interference and candidate compounds requires profound chemical knowledge and experience. It is equally relevant, however, to strive for a data-driven assessment of promiscuity by exploring compound activity data on a large scale ^20^, aiming to identify compounds with interference potential for further analysis. Previously, we have determined that increasing assay frequency of pairs of structural analogs did not correlate with differences in promiscuity ^23^. The current analysis was focused on hit rates of individual compounds that were extensively assayed to identify the overall most active chemical entities. Therefore, we have carried out a statistical analysis of hit rates of compounds that were extensively tested in screening assays. These compounds were evaluated in on average 411 assays (with a median value of 437 assays per compound). A special feature of this study has been its focus on pairs or larger series of analogs, rather than single compounds, which provides additional confidence criteria for activity assessment and further increases the information content of activity data analysis. Many series of analogs with much higher than typically observed hit rates and largely consistent activity profiles across many different assays were identified. This collection of series provides a basis for further investigating compounds with interference potential or true multi-target activities.

## Methods

### Compounds

From the PubChem BioAssay database ^16^, 437,257 compounds were pre-selected that were tested in both primary and confirmatory assays, representing extensively assayed screening compounds ^23^. Approximately 95% of these compounds were evaluated in more than 50 primary and/or confirmatory assays ^23^. Primary PubChem assays report compound activity (e.g., percentage activity) for a single dose, while confirmatory assays are dose-response assays yielding titration curves and IC _50_ values. Our current analysis focused on primary assays, for which much larger data volumes were available than for confirmatory assay. Primary assays also included assays for which no target was specified (such as cell-based assays). In addition to larger volumes, primary assays are more prone to false-positives than confirmatory assays, thus providing an upper-level estimate of compound promiscuity consistent with the goals of our analysis. Assignments of active compounds were taken from each individual assay as reported. Screening parameters such as compound concentration and activity criteria were assay-dependent. For pre-selected compounds, hit rate statistics were determined.

### Matched molecular pairs and series

A matched molecular pair (MMP) is a pair of compounds that are only distinguished by a chemical change at a single site ^24^, termed a chemical transformation ^25^. As an extension of the MMP concept, a matched molecular series (MMS) was defined as the union of all MMP compounds that are only distinguished by chemical modifications at a given site ^26^. Accordingly, an MMS represents a series of analogs sharing a single substitution site. To generate MMPs, exocyclic single bonds in screening compounds were systematically fragmented ^25^ following retrosynthetic fragmentation rules ^27^, yielding so-called RECAP-MMPs ^28^. These MMPs were subject to transformation size restrictions in order to limit chemical changes to modifications typically observed in series of analogs ^29^. An MMS was designated as redundant if it was a subset of a larger MMS or if there was another MMS representing the same series of analogs but having a larger MMP core. For screening compounds with high hit rates, non-redundant MMS were systematically determined.

### MMS parameters

For each MMS, three parameters were calculated. First, the *MMS hit rate (HR)* was obtained from the union of all assays (i.e., the number of unique assays in which one or more analogs were tested in) and assays with activity signals (active assays, i.e., the number of unique assays in which one or more analogs were found to be active). Second, *assay overlap* was determined as the proportion of assays in which all MMS compounds were tested in (shared assays, i.e., the intersection of assays) relative to the union of assays. Third, from assay overlap, *assays with inconsistent activity* were calculated as the proportion of shared assays in which different MMS compounds were active or inactive.

All calculations were carried out using in-house Java scripts and KNIME ^30^ protocols with the aid of the OpenEye ^31^ chemistry toolkit.

## Results and discussion

### Study design

A statistical analysis of hit rates of extensively tested screening compounds is presented taking assay frequency into account. On the basis of the hit rate distribution, ranges of unusually high hit rates were determined. Since the majority of compounds that were active in primary assays were also active in confirmatory assays, a high level of consistency in the assignments of active compounds was observed. From compounds with high hit rates, analog series with single substitution sites (MMSs), i.e., “minimal” chemical modifications within series, were systematically extracted, which provided structural context information and hit rate controls for closely related compounds. For MMSs, different parameters were calculated, making it possible to compare and prioritize these series. The collection of MMSs with high hit rates provides a basis for investigating assay interference candidates, as well as chemical entities with potential multi-target activities.

### Source compounds and assay distribution

**Figure 1.**
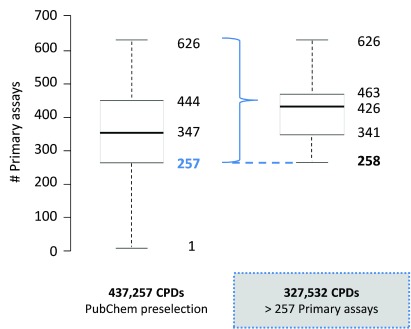
Assay frequency distribution. The frequency distribution of primary assays is shown in a boxplot format for 437,257 pre-selected PubChem compounds and a subset of 327,532 compounds. The plot gives the smallest number of primary assays (lower whisker), first quartile (lower boundary of the box), median value (thick line), third quartile (upper boundary of the box), and largest number of assays (upper whisker). Outliers are not displayed. The dashed blue line indicates the selection criterion for the compound subset (i.e., tested in more than 257 primary assays).

Figure 1 (boxplot on the left) shows the global distribution of primary assays for 437,257 extensively tested PubChem compounds, with a median value of 347 assays per compound. From these, a subset of 327,532 compounds was selected that were tested in more than 257 primary assays, corresponding to the lower quartile boundary of the global distribution. For this subset, the assay distribution was separately monitored ( Figure 1, boxplot on the right), yielding a median of 426 (and a maximum of 626) assays per compound. Hence, half of these compounds were tested in more than 426 primary assays.

### Hit rate distribution

**Figure 2.**
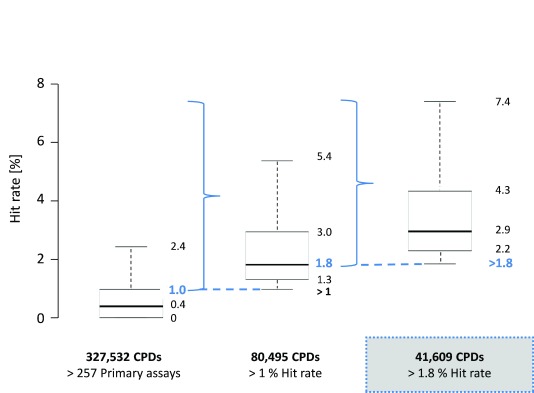
Hit rate distribution. For three different subsets of PubChem compounds, hit rate distributions are shown in boxplots according to Figure 1. The subsets are characterized by increasing hit rates (marked by dashed blue lines).

For 327,532 compounds tested in more than 257 assays, hit rates were determined. The distribution is reported in Figure 2 (boxplot on the left), resulting in a median hit rate of 0.4%. The lower quartile boundary and lower whisker of the boxplot were identical and represented consistently inactive compounds, which were not of interest for our current analysis. On the basis of the distribution, the interval of “bulk hit rates” (b_hr) for these extensively assayed PubChem compounds was defined as 0% < b_hr ≤ 1.0%, covering the lower quartile, median, and upper quartile (and hence the “bulk” of the distribution). There were 80,495 compounds with hit rates ≥ 1.0%. The hit rate distribution of this compound subset is shown in Figure 2 (middle), yielding a median of 1.8%. This value was set as the hit rate threshold for most active screening compounds. The threshold was exceeded by 41,609 compounds, representing 12.7% of the initial compound pool. The hit rate distribution of these compounds is reported in Figure 2 (right), resulting in a median of 2.9%. We determined that 93.1% of the compounds with hit rates greater than 1.8% in primary assays were also active in confirmatory assays (yielding IC _50_ values). Hence, their activity was not confined to primary assays.

### Compound series and parameters

From the 41,609 compounds with highest hit rates, MMSs were systematically extracted on the basis of RECAP-MMPs. After removal of redundant MMSs (see Methods), 6941 unique MMSs were obtained comprising 14,646 compounds, which represented our final hit rate- and series-based selection set. Table 1 reports the size distribution of the MMSs, ranging from two to 17 analogs per series. With 6111 instances, compound pairs and triplets dominated the distribution, but more than 800 larger MMSs were also obtained. Increasing size of MMSs may lead to higher hit rates, variations in assay overlap, and more assays with inconsistent activity. As further discussed below, compound pairs and triplets already provide informative controls for activity analysis and enable a more confident assessment compared to the analysis of individual compounds. This was a major motivation for focusing the analysis on MMSs.

**Table 1.** Size distribution of matched molecular series (MMSs). The distribution of 6941 frequently active MMSs (#MMSs) over increasing numbers of compounds (#CPDs) is reported.

#CPDs	#MMSs
2	4965
3	1156
4	435
5	190
6	70
7	48
8	22
9	21
10	11
11	12
12	3
13	4
14	2
15	1
17	1

**Figure 3.**
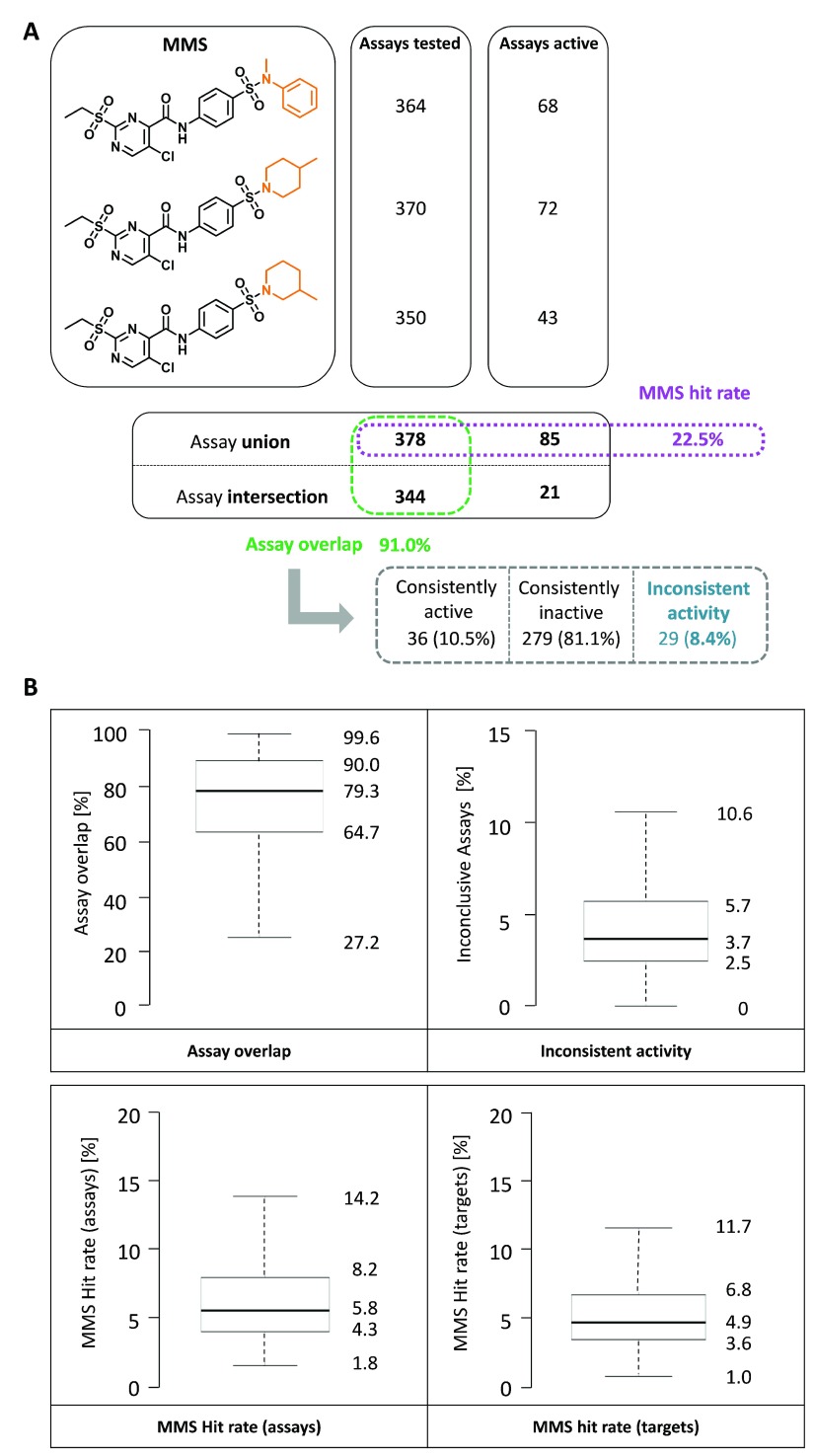
Characterization of matched molecular series (MMSs). ( **a**) An exemplary MMS comprising three analogs is shown. The MMS core and varying substituents are colored in black and orange, respectively. For each compound, the number of assays it was tested and active in is reported, respectively. Furthermore, the assay union, intersection, and MMS hit rate (purple) are given. From these data, the assay overlap (green) of MMS analogs was determined as well as the proportion of assays with consistent activity, inactivity, and inconsistent activity (blue). ( **b**) Boxplots are shown reporting the distribution of assay overlap, assays with inconsistent activity as well as assay- or target-based MMS hit rates for PubChem compounds with greater than 1.8% hit rate.

Figure 3a illustrates the derivation of three parameters for the characterization and comparison of MMSs (rationalized in the Methods section). The cumulative *MMS hit rate* is a direct measure for the activity of a series. In addition, *assay overlap* represents a confidence criterion for MMS assessment, i.e., large assay overlap of compounds comprising a series assigns high confidence to hit rate comparisons. By contrast, the proportion of *assays with inconsistent activity* should best be minimal to draw firm conclusions. Figure 3b reports the distribution of these three parameters for the 6941 MMSs. Assay overlap (upper left plot) and MMS hit rates (lower left) were generally high, with median values of 79.3% and 5.8%, respectively. By contrast, the proportion of inconsistent assays (upper right) was overall low, with a median of only 3.7%. Thus, the distributions of MMS parameters indicated that the set of MMSs was suitable for the analysis of series-based hit rates and hit rate comparison of compounds comprising individual MMSs. We note that MMSs can be ranked in the order of decreasing assay overlap and MMS hit rates and increasing inconsistent assays and prioritized, for example, on the basis of rank fusion calculations.

### Target distribution in primary assays

Our analysis was intentionally focused on hit rates over assays (i.e., assay promiscuity) to take as many activity readouts as possible into account. Therefore, as a control, assay- and target-based hit rates were also compared. Compounds forming the 6941 MMS were evaluated in a total of 1213 assays. For 255 of these assays, no individual target was specified. The remaining 958 assays covered 426 different targets. Figure 3b reports the distributions of MMS hit rates over assays (lower left plot) and targets (lower right). The distributions were overall similar, with median values of assay- and target-based hit rates of 5.8% and 4.9%, respectively. Hence, despite the presence of multiple assays for a subset of targets, assay-based hit rates were only slightly higher than target-based rates, indicating that corresponding conclusions would be drawn from the analysis of these distributions.

### Known interference candidates

The computational aggregation advisor ^4^ and compound strings taken from PAINS filters ^32, 33^ ( http://www.rdkit.org) were used to search the MMSs for known assay interference candidates. The 14,646 MMS compounds contained 783 aggregators (on the basis of 100% similarity) and 2381 compounds with PAINS substructures. There were 611 MMSs with one or more aggregators, 1139 MMSs with one or more PAINS, and 126 MMSs including aggregators and PAINS. However, 5065 MMSs with high hit rates did not contain known compounds with aggregation potential or PAINS substructures. Thus, the MMSs provide a large source of analogs for the exploration of other interference candidates, as well as compounds with true multi-assay/target activities.

### Exemplary series

**Figure 4.**
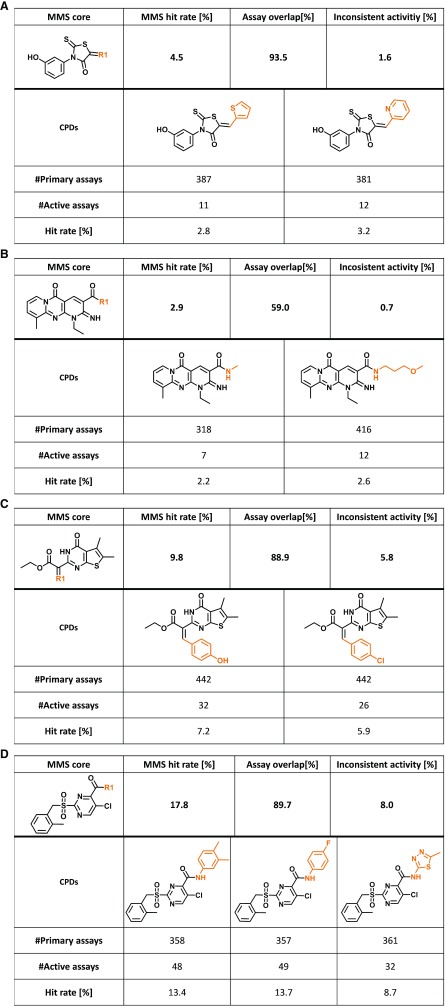
Exemplary matched molecular series (MMSs). ( **a**– **d**) Four exemplary MMSs (core, black; substituents, orange) are shown and the MMS hit rate, assay overlap, and proportion of assays with inconsistent activity are reported. In addition, for each individual analog, its assay frequency and hit rate are provided.

Figure 4 shows exemplary compound pairs and triplets with high assay promiscuity. The two analogs in Figure 4a were tested in more than 380 assays with 93.5% assay overlap and only 1.6% inconsistent assays, yielding comparable hit rates of 2.8% and 3.2%, respectively, resulting in an MMS hit rate of 4.5%. These analogs contained a classical PAINS substructure (ene_rhodanine) ^8, 9^. Furthermore, compounds in Figure 4b were analogs of a molecule with aggregation potential ^4^. They were tested in more than 300 and 400 assays, respectively, yielding a relatively low assay overlap of 59%, and had hit rates of 2.2% and 2.6%, respectively, resulting in a low MMS hit rate of 2.9%. Thus, these analogs were far from being consistently active, as one might assume for strong aggregators. In Figure 4c, a pair of thieno[2,3-d]pyrimidine-2-acetic acid ethyl ester analogs is shown that were tested in 442 assays with large overlap. These compounds had high hit rates of 5.9% and 7.9%, respectively, resulting in a high MMS hit rate of 9.8%. Moreover, Figure 4d shows a triplet of sulfonylpyrimidines that were tested in 357–361 assays with 89.7% overlap, having very high hit rates of 8.7% (one analog) and more than 13% (two analogs). The analogs in Figure 4c and Figure 4d have previously not been classified as interference candidates. The interference potential of sulfonylpyrimidines was assessed via a SciFinder substructure search for 2-[(phenylmethyl)sulfonyl]-pyrimidine. This substructure appeared in more than 100 publications related to biological studies and more than 1000 chemical reactions. Although the potential of sulfonylpyrimidines to undergo nucleophilic aromatic substitutions in organic synthesis is well established in the literature ^34, 35^, reactivities under assay conditions remain to be confirmed experimentally. Compounds forming each of the MMSs in Figure 4 displayed consistent hit rate characteristics, hence assigning confidence to their observed activity phenotype. Taken together, these examples of analog pairs and triplets (i.e., minimally sized MMSs) are indicative of the potential of well characterized MMSs for follow-up investigations focusing on assay interference and multi-target activities.

## Conclusions

Herein, a detailed analysis of hit rates of nearly 440,000 extensively assayed screening compounds has been presented. On the basis of hit rate distributions, 12.7% of the compounds with highest hit rates were selected. From these compounds, analog series with single substitution sites were systematically extracted to complement hit rate statistics with the assessment of structural relationships between active compounds. A total of 6941 unique MMSs were obtained comprising 14,646 compounds. These MMSs were characterized using different parameters prioritizing high-confidence series for activity analysis. A major goal of our study has been the data-driven generation of a pool of analog series for the evaluation of assay interference potential and multi-target activities. More than 5000 MMSs did not contain known interference candidates, providing an opportunity to evaluate compounds with interference potential on a large scale. In the next step, analog series will be evaluated from a medicinal chemistry perspective to complement and further extend statistical considerations. Annotated series and associated assay/target information will then be made freely available. The statistics and selection steps reported herein also make it possible to regenerate compound subsets at different hit rate levels and subject them to further analysis. In addition, large numbers of compounds with high hit rates that were not part of MMSs are also available. For reasons discussed, our preferred approach is taking compound series information into account when judging assay promiscuity.

## Data availability

The data referenced by this article are under copyright with the following copyright statement: Copyright: © 2017 Gilberg E et al.

Data associated with the article are available under the terms of the Creative Commons Zero "No rights reserved" data waiver (CC0 1.0 Public domain dedication). 

The data sets used in this study are freely available in PubChem and can be generated following the selection protocol reported in the Methods.
