# Supplementary material for: Towards a systematic assessment of assay interference: Identification of extensively tested compounds with high assay promiscuity
Source: F1000Res. 2017 Oct 9;6:Chem Inf Sci-1505. Originally published 2017 Aug 17. [Version 2] doi: 10.12688/f1000research.12370.2 (PMC5596351; doi:10.12688/f1000research.12370.2)
Supplement: Peer review history

# Peer review: Referee response for version 2

**DOI:** 10.5256/f1000research.13934.r26808

**Published:** 2017-10-10

**Authors:** John A. Lowe III

**Affiliations:** 1 Jl3pharma LLC, Stonington, CT, USA

I have read this submission. I believe that I have an appropriate level of expertise to confirm that it is of an acceptable scientific standard.

# Peer review: Referee response for version 2

**DOI:** 10.5256/f1000research.13934.r26809

**Published:** 2017-10-10

**Authors:** José L Medina-Franco, Fernanda I. Saldívar-González

**Affiliations:** 1 Department of Pharmacy, Faculty of Chemistry, National Autonomous University of Mexico, Mexico City, Mexico

The authors have addressed the minor points raised by the referees.

We have read this submission. We believe that we have an appropriate level of expertise to confirm that it is of an acceptable scientific standard.

# Peer review: Referee response for version 1

**DOI:** 10.5256/f1000research.13396.r25426

**Published:** 2017-10-02

**Authors:** Michael Walters

**Affiliations:** 1 Department of Medicinal Chemistry, University of Minnesota, Minneapolis, MN, USA

This is an excellent manuscript that will certainly help researchers further understand the liabilities of "good-actors"; compounds that provide structure-interference relationships (SIR) that may not be pertinent to the biology being studied.

 Notes:

1. Inconsistent is misspelled in Panel B of Figure 3.
2. Though this will need to be experimentally-verified, the sulfonylpyrimidines are almost certainly interfering by SnAr reactions. A literature search to gauge this potential reactivity of the core structure retrieved >100 articles and >1000 reactions. This group is certainly not in the PAINS definitions, but its assay interference potential can readily be ascertained by a quick reaction substructure search. Most importantly, this observation reinforces that lack of PAINS "flagging" is not sufficient to ensure that compounds don't have assay interference potential. The authors may wish to highlight this in their discussion.

I have read this submission. I believe that I have an appropriate level of expertise to confirm that it is of an acceptable scientific standard.

# Peer review: Referee response for version 1

**DOI:** 10.5256/f1000research.13396.r25674

**Published:** 2017-09-12

**Authors:** José L Medina-Franco, Fernanda I. Saldívar-González

**Affiliations:** 1 Department of Pharmacy, Faculty of Chemistry, National Autonomous University of Mexico, Mexico City, Mexico

The manuscript addresses a timely topic and is part of an effort to understand the multitarget activities of small molecules and their potential utility or drawbacks for drug discovery.

 Using the MMs formalism, the authors developed a protocol to identify analog series with high hit rates. The MMs approach responds to the need to generate SAR information of interference compounds. This approach allows for more reliable evaluation compared to the analysis of individual compounds.

 The analogue series identified with data from PubChem should be useful for medicinal chemistry programs. Similarly, the protocol developed in this work using MMPs should be useful to mine other large screening data sources (either public or proprietary data sets).

 Minor suggestions to further improve the quality of the manuscript:

- Comment on the manuscript the effect of the compound concentration that is used to define a “hit” compound in a given assay. In other words, since it is unlikely that the same compound concentration is used across all assays, how this variable influences the “hit rates” and conclusions of the study?
- The current analysis is made based primarily in primary assays in PubChem. In the Methods authors justify that there are larger data volumes for primary assays. We agree but would be nice to see in the manuscript a comment regarding the balance between volume vs. quality of the data.
- We found it very pertinent that the study focused on compounds with high rates in primary assays, since more than 90% of these compounds are also active in confirmatory trials. It is interesting to comment how the hit rate is related to the quality of the data.
- An earlier study ^1^ mentions that the assay frequency is not correlated with increased promiscuity. It is desirable to include a commentary in the manuscript when discussing the frequency of the test distribution and the generation of the first subset of 327,532 compounds.
- Three parameters for the characterization and comparison of MMSs were determined (hit rate (HR), assay overlap, assays with inconsistent activity). Does the MMS size affect these determinations?

Other suggestions (per section):

- Introduction, last paragraph:  briefly summarize the major findings of previous works related to this study (e.g., refs. 18, 19, 23) and emphasize the novelty of this work.
- Shorten the title (and include the concept of MMPs).
- In the Introduction (last paragraph), include figure numbers for expressions such as “extensively”, “larger’, “many”, “much higher”. For instance, when the authors mention “extensively tested” in the title and through the manuscript, they mean “>10,000”, “>100,000”, “>400,000”, etc.
- Methods: elaborate a bit more on the in-house scripts, e.g., the program language.

We have read this submission. We believe that we have an appropriate level of expertise to confirm that it is of an acceptable scientific standard.

## References

[1] HuYBajorathJ: Entering the 'big data' era in medicinal chemistry: molecular promiscuity analysis revisited. *Future Sci OA*.2017;3(2) : 10.4155/fsoa-2017-0001 FSO179 10.4155/fsoa-2017-0001 28670471PMC5481856

# Peer review: Referee response for version 1

**DOI:** 10.5256/f1000research.13396.r25140

**Published:** 2017-08-18

**Authors:** John A. Lowe III

**Affiliations:** 1 Jl3pharma LLC, Stonington, CT, USA

This article makes an important contribution to addressing a serious complication in screening for new biological activity, especially in the context of new drug discovery.  Several researchers continue to point out the waste of time and money in following up on false positive hits from biological screening programs.  While paradigms to filter out false positives, such as aggregators or other “frequent hitters” such as PAINs, are becoming mainstream techniques, these may not be sufficient, either because they miss some false positives or because they mistakenly classify legitimate hits as false positives.  By carrying out a statistical analysis of a large set of screening data for hit rates using matched molecular pairs and series, this article offers a valuable perspective on this issue.

 There are several aspects of this article that are particularly valuable.  For example, Figure 3b is an important control showing that using assay hit rate does not overstate promiscuity, in that target hit rate is similar, linking the results to a biological mechanism of action.  In Figures 4c and 4d, the exemplified compounds appear to be Michael acceptors, and could react with Cys residues covalently, or sequester thiol reagents used in the assay, which could explain their promiscuity.  It is worth pointing these out, as they would not be picked up in PAINs filters and only by a knowledgeable chemist.  Overall, this work is as important contribution to the ongoing effort to reduce the occurrence of false positive hits serving as starting points for discovery programs.

I have read this submission. I believe that I have an appropriate level of expertise to confirm that it is of an acceptable scientific standard.
